# Predicting and mapping human risk of exposure to *Ixodes ricinus* nymphs using climatic and environmental data, Denmark, Norway and Sweden, 2016

**DOI:** 10.2807/1560-7917.ES.2019.24.9.1800101

**Published:** 2019-02-28

**Authors:** Lene Jung Kjær, Arnulf Soleng, Kristin Skarsfjord Edgar, Heidi Elisabeth H Lindstedt, Katrine Mørk Paulsen, Åshild Kristine Andreassen, Lars Korslund, Vivian Kjelland, Audun Slettan, Snorre Stuen, Petter Kjellander, Madeleine Christensson, Malin Teräväinen, Andreas Baum, Kirstine Klitgaard, René Bødker

**Affiliations:** 1 Department for Diagnostics and Scientific Advice, National Veterinary Institute, Technical University of Denmark, Lyngby, Denmark; 2 Department of Pest Control, Norwegian Institute of Public Health, Oslo, Norway; 3 Department of Virology, Norwegian Institute of Public Health, Oslo, Norway; 4 Department of Production Animal Clinical Sciences, Norwegian University of Life Sciences, Oslo Norway; 5 Department of Natural Sciences, University of Agder, Kristiansand, Norway; 6 Sørlandet Hospital Health Enterprise, Research Unit, Kristiansand, Norway; 7 Department of Production Animal Clinical Sciences, Section of Small Ruminant Research, Norwegian University of Life Sciences, Sandnes, Norway; 8 Department of Ecology, Wildlife Ecology Unit, Swedish University of Agricultural Sciences, Grimsö, Sweden; 9 Department of Applied Mathematics and Computer Science, Technical University of Denmark, Lyngby, Denmark

**Keywords:** *Ixodes ricinus*, tick-borne disease, northern Europe, exposure risk, public health, climate, boosted regression trees, environmental satellite data, human population density

## Abstract

**Background:**

Tick-borne diseases have become increasingly common in recent decades and present a health problem in many parts of Europe. Control and prevention of these diseases require a better understanding of vector distribution.

**Aim:**

Our aim was to create a model able to predict the distribution of *Ixodes ricinus* nymphs in southern Scandinavia and to assess how this relates to risk of human exposure.

**Methods:**

We measured the presence of *I. ricinus* tick nymphs at 159 stratified random lowland forest and meadow sites in Denmark, Norway and Sweden by dragging 400 m transects from August to September 2016, representing a total distance of 63.6 km. Using climate and remote sensing environmental data and boosted regression tree modelling, we predicted the overall spatial distribution of *I. ricinus* nymphs in Scandinavia. To assess the potential public health impact, we combined the predicted tick distribution with human density maps to determine the proportion of people at risk.

**Results:**

Our model predicted the spatial distribution of *I. ricinus* nymphs with a sensitivity of 91% and a specificity of 60%. Temperature was one of the main drivers in the model followed by vegetation cover. Nymphs were restricted to only 17.5% of the modelled area but, respectively, 73.5%, 67.1% and 78.8% of the human populations lived within 5 km of these areas in Denmark, Norway and Sweden.

**Conclusion:**

The model suggests that increasing temperatures in the future may expand tick distribution geographically in northern Europe, but this may only affect a small additional proportion of the human population.

## Introduction

Ticks are one of the most important vectors for pathogens, impacting a wide range of vertebrates, and transmit more pathogens than any other arthropod [1,2]. In Europe, the main vector for tick-borne pathogens is *Ixodes ricinus* [3,4], which is also the most common tick species in Scandinavia [3-5]. Over the last decades, the incidence and geographical range of tick-borne diseases have increased [3,6,7] and pose a risk to both human and animal health. Scandinavia constitutes the edge of the northern distributional range of *I. ricinus* [4]. The incidence of Lyme borreliosis (LB) and tick-borne encephalitis (TBE) is increasing in both Norway and Sweden [5,8-10]. In Norway, LB and TBE have mostly been reported along the coastline in the southern parts of the country [5,8]. However, tick-borne encephalitis virus (TBEV) has been found in *I. ricinus* nymphs as far north as ca 115 km from the Arctic Circle [11,12]. In Sweden, LB is widespread in the southern and eastern regions [4,13,14], whereas TBE is concentrated in the south-central and coastal regions, with the annual TBE incidence around Stockholm exceeding 4 per 100,000 inhabitants [9,10,15,16]. In Denmark, LB seems endemic and widespread [3], whereas TBEV-infected ticks have only been confirmed on the island of Bornholm and at one emerging site in northern Zealand with two human cases [17,18].

The increase in incidence and geographical range of pathogens and their tick vector is likely to be a combination of several factors, e.g. climate and availability of host species [6,7], which all affect the ticks’ life cycle and therefore their distribution and the possibility of tick-borne diseases being present in specific regions [7,19]. Many hard ticks, as *I. ricinus*, are sensitive to climate and weather [1,6], and are restricted to live in areas with high rainfall and vegetation that keeps a humidity of at least 80%, to prevent desiccation when the ticks are off-host [1,7]. Knowing the distribution of ticks may help pinpoint potential risk areas for disease transmission and guide health authorities in determining where to focus surveillance efforts, where to use preventive measures, or where to put emphasis on informing people.

Determining tick distribution can be a difficult task depending on the size of the area of interest. Throughout Scandinavia, there have been several field studies on ticks and their associated pathogens [3,8,12,20-24], but in order to predict tick presence in unsampled regions in the present but potentially also for the future, we need repeatable survey methods and to find factors associated with tick abundance that can aid us in developing models with high predictive power. In Norway, Jore et al. [2] used sheep serum antibody-positive for tick-borne *Anaplasma phagocytophilum* as a proxy for tick presence, finding effects of temperature, abundance of large cervids and farm animals as well as land cover on tick distribution. Studies in Sweden found significant effects of climate, vegetation parameters and length of vegetation period on tick abundance and distribution [13,14]. In Denmark, Jensen [23] found that *I. ricinus* nymph abundance was significantly affected by the interaction between soil water capacity and the number of hunted roe deer. Several other studies from Europe and North America have also found a link between environmental factors and tick distribution, such as temperature, vegetation indexes and vapour pressure [25-27].

Although climate, land cover and host abundance may all play a role in tick distribution, it can often be difficult to obtain extensive data on host species, whereas environmental, weather and climate data are more readily available from satellite images and weather models. Machine learning techniques are increasingly used in developing models for vector predictions as they are flexible, can account for nonlinearity and interactions and can handle different types of predictor variables, such as satellite images of environmental data [28,29]. Machine learning techniques combined with environmental predictors have been used in modelling biting midges (*Culicoides* sp.) [30-33], and mosquitoes [28,34,35], and studies on ticks include modelling tick distribution or abundance [36-38] as well as the distribution of tick-borne human diseases [15,39].

The risk of human exposure to ticks, and potentially tick-borne diseases, depends on tick and host dynamics as well as human behaviour [40]. Several studies have reported that living in areas in close proximity to forest increases the risk of LB or TBE [41-43] as *I. ricinus* is more abundant in forest habitats [21,40].

We here present a novel map of nymphal *I. ricinus* distribution for Scandinavia using machine learning algorithms applied to field data, collected in a strict standardised design in the period from 15 August to 30 September 2016. Furthermore, we relate our modelling results of tick distribution to public data on human population density and to the distance to the predicted suitable tick habitats, in order to assess the potential public health impact.

## Methods 

### Stratification of study region and site selection

**Figure 1:**
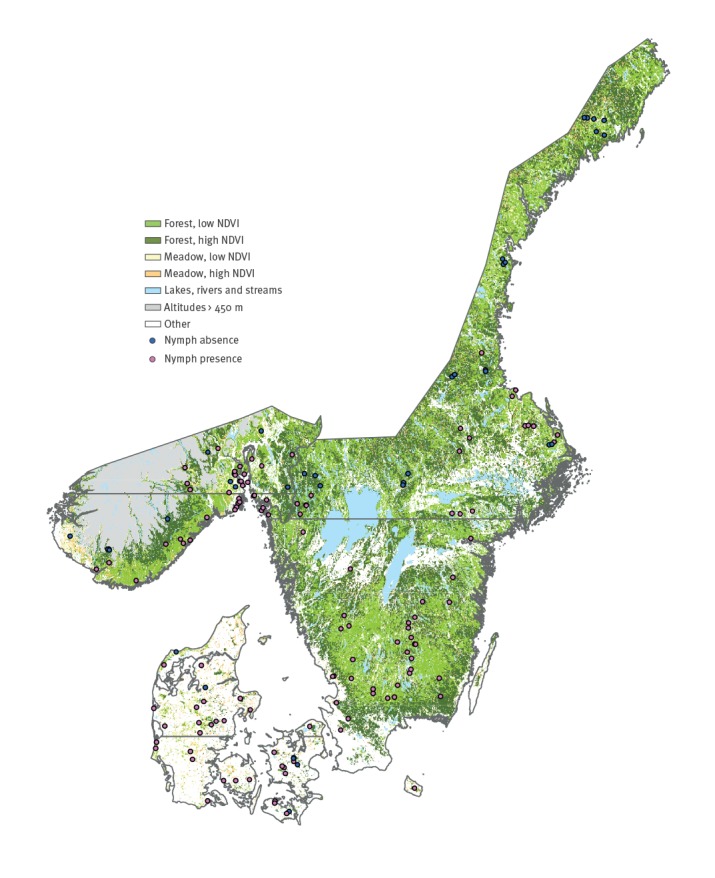
Stratification of the study area, showing 159 sample sites and presence/absence of *Ixodes ricinus* nymphs, Denmark, Norway and Sweden, 15 August–30 September 2016

This study was part of a larger study, where additional objectives were to measure tick abundance and collect nymphs for pathogen detection in Denmark, Norway and Sweden. The field collection region for *I. ricinus* nymphs was for logistical reasons limited to 274,660 km^2^ including all of Denmark, southern Norway and southern Sweden as well as the Swedish eastern coastal zone (Figure 1). Within this area, we excluded all altitudes of 450 m above sea level and higher (19,926 km^2^), where ticks are rare or absent [5]; these altitudes were also excluded from the final prediction map.

NDVI: normalised difference vegetation index.

Forest includes the cover types: broad-leaved forest, coniferous forest and mixed forest. Meadow includes: land principally occupied by agriculture with significant areas of natural vegetation, natural grasslands, moors and heathland, and transitional woodland-shrub. The lines divide each country into equally sized northern and southern strata. Only parts of Norway and Sweden were included in the field study for logistic reasons.

We stratified the remaining land area (234,191 km^2^, excluding lakes and waterways) using Fourier processed satellite imagery of the normalised difference vegetation index (NDVI) [44] and Corine land cover data (1 km^2^ resolution) [45] to define forest and meadow habitats. Other land cover categories were not sampled for ticks and were left out of the prediction map. For details about the stratification and Fourier-processed satellite imagery, see the Supplement.

We randomly selected 30 first-priority sample sites (80% forest and 20% meadow, Supplementary Table S2) in each of the three countries (R 3.4.2 [46] and sampleStratified in the raster package). This number was logistically the maximum number of sites feasible to visit within a reasonable timeframe. We decided to collect 80% of the samples from forested areas, as forest areas are the most important tick habitat [21,40]. Furthermore, 10 alternative sites for each first-priority site were randomly selected in the same stratum and ordered in priority after shortest distance to original site. These alternative sites were created in case of problems with access to the priority area or difficulties collecting nymphs (for pathogen detection). If a priority area could not be sampled, we would move on to the first alternative site and so forth, keeping the abundance data from the original site if available. For each meadow site, we additionally created 10 alternative forest sites, to be sampled should it prove impossible to collect ticks in meadows.

Because we were interested in investigating tick abundance along the Oslo Fjord in detail, we chose a further 20 random sites along the fjord (maximum distance of 800 m from the coast), with 10 alternatives for each of the 20 sites (same setup as above, Supplementary Table S3).

### Field study

For logistical reasons, we conducted the field study between 15 August and 30 September 2016. We measured tick abundance during the day between 11:00 and 16:00, using a 100 m north- and a 100 m east-facing transect, meeting at a 90° angle at one end. We sampled for questing *I. ricinus* ticks by dragging a white flannel cloth (1.05 × 1.15 m, containing lead weights at one end) 100 m along each transect, turning and dragging it 100 m back; we removed and counted larvae, nymphs, adult male and adult female ticks every 50 m. As some sites had very low abundance of nymphs or none, an alternative site with lower priority was chosen for nymph collection, while keeping abundance data from the original sites, thus resulting in a different number of sites with abundance measures per country. If one or more nymphs were found on the two transects, the site was classified as ‘nymph presence’ else it was classified as ‘nymph absence’.

### Presence/absence modelling

We developed a boosted regression tree (BRT) prediction model on the presence/absence data for nymphs, using 92 environmental predictors (Table 1). BRT is a machine learning technique based on two algorithms: regression trees and gradient boosting [29]. This technique allows predictions of a response variable, in our case presence/absence. The estimated probability of presence (PP) can then be plotted as a risk map with a resolution of 1 km^2^. For additional details regarding the environmental predictors, the BRT method used, balancing of the data and cross validation of the model, see the Supplement. 

**Table 1:** Environmental predictors used in the boosted regression tree models to predict probability of the presence of *Ixodes ricinus* nymphs in the modelled Scandinavian region, Denmark, Norway and Sweden, 15 August–30 September 2016

Source	Variables
Modis (Fourier transformed), 2001–12^a^ [44]	Middle infra-red
	Daytime land surface temperature
	Night-time land surface temperature
	Normalised difference vegetation index (NDVI)
	Enhanced vegetation index (EVI)
WorldClim 1.4, 1960–90 [49]	Altitude
BioClim (WorldClim), 1960–90 [49]	BIO1: Annual mean temperature
	BIO2: Mean diurnal range (mean of monthly (max–min temperature))
	BIO3: Isothermality (BIO2/BIO7) × 100
	BIO4: Temperature seasonality (standard deviation × 100)
	BIO5: Max temperature of warmest month
	BIO6: Min temperature of coldest month
	BIO7: Temperature annual range (BIO5–BIO6)
	BIO8: Mean temperature of wettest quarter
	BIO9: Mean temperature of driest quarter
	BIO10: Mean temperature of warmest quarter
	BIO11: Mean temperature of coldest quarter
	BIO12: Annual precipitation
	BIO13: Precipitation of wettest month
	BIO14: Precipitation of driest month
	BIO15: Precipitation seasonality (coefficient of variation)
	BIO16: Precipitation of wettest quarter
	BIO17: Precipitation of driest quarter
	BIO18: Precipitation of warmest quarter
	BIO19: Precipitation of coldest quarter
Harmonized World Soil Database v 1.2 (FOA, IIASA), 2009 [50]	Soil types, depicted by Soil Mapping Unit Code of major soil group (FAO-90 soil classification system)
Gridded Population of the World Dataset (SEDAC), 2015 [47]	Population counts per 1 km^2^

^a^ For each variable, the Fourier processing output includes mean, minimum, maximum, variance in raw data, combined variance in annual, bi-annual, and tri-annual cycles as well as amplitude, phase and variance of annual, bi-annual and tri-annual cycle.

All predictors come as raster files with a resolution of 1 km^2^.

The MODIS-derived data (Table 1) stem from time series data (12 years), whereas our field sampling only occurred in the year 2016. However, at any given time, the abundance and presence of *I. ricinus* instars are influenced by environmental conditions in previous years (adult females surviving to lay eggs, survival of eggs during winter, prolonged diapause of nymphs and larvae) and are not just dependent on the environmental conditions in the collection year. Time series data provide us with data on seasonality and the potential range of the environmental variables, allowing us to make more general predictions on *I. ricinus* distribution in southern Scandinavia.

### Human risk of tick exposure

After identifying a final prediction map, we used the Gridded Population of the World dataset (raster with 1 km^2^ resolution [47], Table 1), to identify the number of people living in areas within various distances to forest and meadows where the PP was higher or equal to 10%, 20%, 30%, 40%, 50%, 60%, 70%, 80% and 90%. We chose distances from 1 km to 5 km to depict people living in close proximity to potential tick habitats. Details can be found in the Supplement.

## Results

### Field study

We measured tick abundance at 37 sites in Denmark, 75 sites in Sweden and 47 sites in Norway. The 159 sites constitute 63.6 km of dragged transects (Table 2, Figure 1).

**Table 2:** Number of sites surveyed and data on presence/absence of *Ixodes ricinus* nymphs, Denmark, Norway and Sweden, 15 August–30 September 2016

Country	Total number of sites surveyed	Number of sites with presence of *Ixodes ricinus* nymphs	Number of sites with absence of *Ixodes ricinus* nymphs
Denmark	37	32	5
Norway	47	38	9
Sweden	75	55	20

### Presence/absence modelling

**Figure 2:**
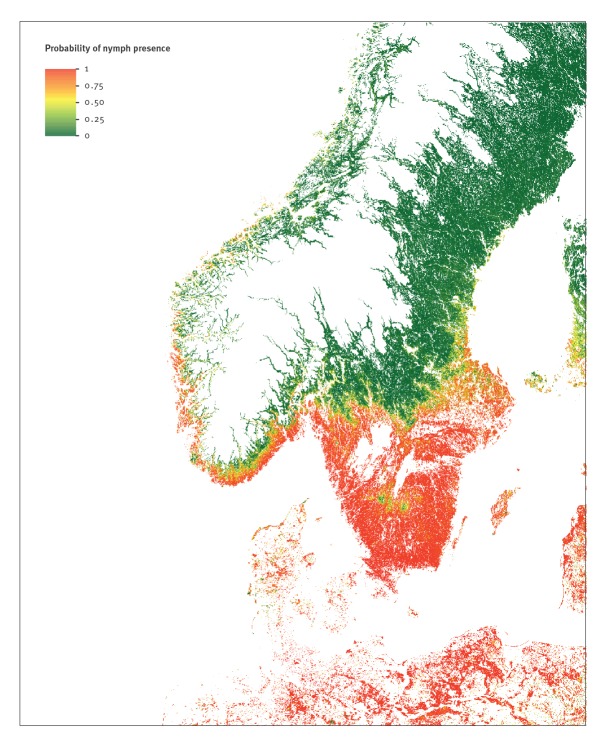
Predicted probability of presence of nymphal *Ixodes ricinus*, produced by the final boosted regression tree model, Denmark, Norway and Sweden, 15 August–30 September 2016

The final BRT model had an accuracy of 0.85, a sensitivity of 91% and a specificity of 60% (given a fixed cut-off of 50% PP). The area under the curve for the receiver operating characteristic was 0.86 (Supplementary Figure S2) [29]. As specificity was only 60% (with the default PP cut-off of 50%), we plotted the prediction errors (observed data – mean predicted probability of presence over the folds and the repeats) in order to visualise a potential spatial pattern (Supplementary Figure S3). From the spatial map, we concluded that the low specificity was mainly due to sites in Denmark and Norway (close to the Swedish border). The final prediction map encompassed 100%, 68.4% and 85.8% of Denmark, Norway and Sweden’s total land area, respectively (Figure 2). We only made predictions for forest and meadow habitats that corresponded to our sampling sites. Habitats with at least 50% PP of tick nymph presence (17.5% of the total modelled area) constituted 15.7% of Denmark’s, 7.4% of Norway’s and 23.9% of Sweden’s land area within the modelled region. Assuming that tick presence in the areas of northern Norway and Sweden not included in the modelled region was below 50% PP, the percentage of a predicted tick risk of at least 50% was 5.1% and 20.5% of the total land area of Norway and Sweden, respectively.

This map depicts the predicted region (100%, 68.4% and 85.8% of Denmark’s, Norway’s and Sweden’s total land area). White areas within Denmark, Norway and Sweden are altitudes above 450 m or lakes, rivers and streams, or habitats other than our sampled forest and meadow habitats (not predicted).

The most important predictors in the final model were day- and night-time land surface temperatures and other parameters related to temperature, land cover (lower PP in transitional woodland-shrub compared with the other cover types), the middle infrared index and related parameters, and parameters related to the vegetation indices enhanced vegetation index (EVI) and NDVI (see plots of the top 5 predictors, Supplementary Figure S4). 

### Human risk of tick exposure

**Figure 3:**
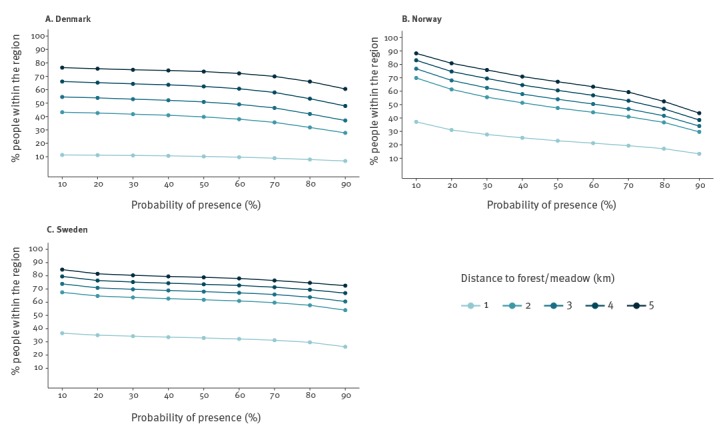
Percentage of people in the predicted region living within 1, 2, 3, 4 and 5 km of forest and meadow with different cut-offs for probability of presence of nymphal *Ixodes ricinus*, Denmark, Norway and Sweden, 15 August–30 September 2016

**Figure 4:**
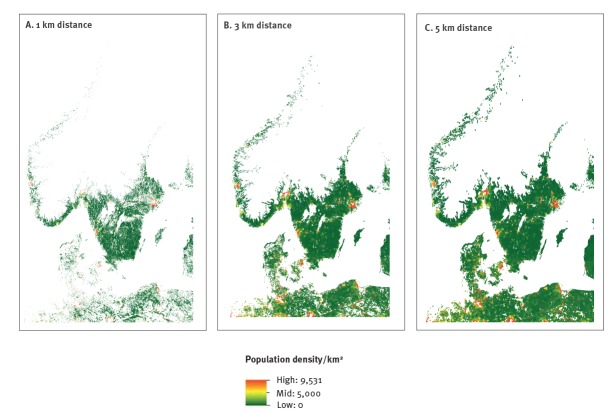
Areas with people living at different distances to forest/meadow that have a probability of presence of nymphal *Ixodes ricinus* of at least 50%, Denmark, Norway and Sweden, 15 August–30 September 2016

The modelled region incorporating all altitudes included 19.4 million people, with 5.5 million (28.4%), 4.5 million (23.2%) and 9.4 million (48.5%) in Denmark, Norway and Sweden, respectively, which corresponded to 100% of the total Danish population, 91% of the total Norwegian population and 97% of the total Swedish population (based on the population density raster file). The proportion of people living within 1 km of forest and meadow was consistently lower for Denmark (ranging from 11% to 7% with increasing PP) than for Norway (ranging from 37% to 13% with increasing PP) and Sweden (ranging from 37% to 26% with increasing PP) for all PP values (Figure 3). This number increased consistently as distance to forest and meadow reached 5 km with 76–61%, 88–44% and 85–73% of the regional population living within 5 km of forest or meadow with PP values ranging from 0.1 to 0.9 for Denmark, Norway and Sweden, respectively (Figure 3). Figure 4 depicts areas where people live within 1, 3 and 5 km of forest or meadow for a fixed PP value of 50%.

## Discussion

Using the machine learning technique Boosted Regression Trees, we were able to create maps of the probability of nymphal *I. ricinus* presence in Scandinavia with high predictive power based on a standardised repeatable procedure. The predicted distribution corresponded well with what is generally believed about tick distribution in Scandinavia, assuming that a PP lower than 50% is a true absence. The higher probabilities of presence around the southern Norwegian coast line is in agreement with the distribution maps known for Norway [5,24]. In Sweden, we found higher PPs in the southern parts, with a boundary north of the large lakes, above which PP was low. This border coincides well with the biogeographical and climatic boundary called Limes Norrlandicus (LN) that separates the species-rich boreo-nemoral zone with shorter and milder winters in the south, from the boreal zone in the northern parts of Sweden [48]. Before the 1980s, LN used to be the range limit for *I. ricinus* in Sweden [4], but since then, the range of *I. ricinus* has expanded beyond this biogeographical border albeit at low abundances [4]. Our model reflected this pattern, showing higher PP below LN and a quick drop in PP above LN, but with a low PP throughout this northern region. The distinct patches of low PP below the great lakes in Sweden follow observed lower temperatures at these two elevated areas (Supplementary Figure S5). The PP was high throughout Denmark, except for the dry heathlands and sandy habitats of central and western Jutland. This pattern corresponds well with what we know about tick biology and the need for a high relative humidity to sustain ticks in a given habitat [7].

Our model had low specificity compared with the sensitivity. Since the main priority of this study was prediction of true presences, we refrained from increasing the specificity, which could have been obtained by choosing a higher cut-off value than the fixed 50%. In general, certainty of true absence can be hard to obtain, as presence/absence is always dependent on the sampling effort. Our recorded absences may not have been true absences and our model may still have predicted presence based on the environmental variables for that specific site. Conversely, high local abundance of deer hosts may facilitate establishment of ticks in areas for which the model predicted absence. In our data, we had a low proportion of absences (21%) and for Denmark alone, this number was 15.6%. Even though we used balancing methods to account for this disproportion, it is possible that our empirically collected sample could not feed the model with enough absence data to learn how to accurately predict absences, thus resulting in low specificity.

We were able to create a model with high predictive power based on environmental predictors. We found that land surface temperatures as well as measures of high vegetation cover (middle infrared light is absorbed by leaves and vegetation, thus densely vegetated areas reflect less middle infrared light) positively influenced the probability of nymph presence. However, the resulting modelled distribution may be due to other environmental factors correlated with these predictors, such as the climatic impact on vegetation and host species. Although ticks can be directly affected by temperatures and humidity [1,4,6,7], they are also dependent on their host species for survival and dispersal [4,7,9]. Abundance of host species may in turn be directly and indirectly affected by climate and weather [4,7,13], thus making it hard to separate factors into causal and confounding. Despite lacking fine resolution data on host abundance, we were able to use environmental predictors to create a biologically plausible model for *I. ricinus* presence/absence in Scandinavia.

Overlaying our distribution maps for tick nymphs with human population density maps revealed the proportion of people potentially at risk for tick exposure. Based on studies estimating the risk of LB or TBE in relation to landscape characteristics around residential homes [41-43], we set the maximum distance from forest or meadow to 5 km. In general, we found that a large percentage of the population in the region live within 5 km of forest and meadows with a risk of tick presence, even if we set the cut-off for PP to be higher than the default 50%. Particularly for Norway, our model predicted high probability of nymph presence only for a very small area around the coast line; with a 50% PP cut-off, this area amounted to just 5.1% of Norway’s total land area. Whereas this small area seems negligible, human population densities in Norway are relatively higher in these areas, exposing more people to tick habitats than we would expect by looking at the area alone, as 67% of the Norwegian population live within 5 km of forest and meadow with PP ≥ 50%. That changing the PP cut-off value had a larger effect on the percentage of people at risk in Norway compared with Denmark and Sweden is probably due to a steep temperature gradient as we move away from the coast, caused by elevation-dependent temperatures (Supplementary Figure S5).

In the United States, Glass et al. [42] found that the odds of contracting LB increased within ca 1 km of living close to forested habitats. The proportion of people living within 1 km of forest or meadow is particularly low for Denmark no matter the PP cut-off (11–7%). This may however be a gross underestimation of exposure risk as Denmark has many fragmented small forest patches interspersed with agricultural fields and urban areas and these small patches may not show up in our coarse resolution of 1 km^2^. However, little is known about how likely these non-sampled areas are as tick habitats. In Norway and Sweden, a higher proportion of the population (between 37% and 13% at different PP cut-off values) are living within 1 km of forest or meadow.

This study showed that given the current distribution of ticks in Scandinavia, a high percentage of inhabitants are already exposed to the risk of tick bites (within a distance of 5 km to forest or meadow with a 50% PP, respectively 73.5%, 67.1% and 78.8% of the Danish, Norwegian and Swedish population may be at risk). The northward expansion of ticks and tick-borne pathogens in Norway and Sweden is a considerable public health concern [9]. However, human population densities in northern Norway and Sweden are low compared with the southern regions, and a tick range expanding north will therefore affect a smaller proportion of the human population. Our results therefore suggest that it may be desirable to target our surveillance and preventive measures in areas with high human population density and where ticks are well established, i.e. the whole of Denmark, the southern coastal parts of Norway, southern Sweden and Sweden’s densely populated eastern coast along the Bothnian Bay.

Machine learning techniques allowed us to produce models and maps with high accuracy and predictive sensitivity for the whole region without having to sample every habitat. These models have highlighted areas at high risk of tick exposure and thus potentially of vector-borne diseases, and can help in targeting these areas for costly surveillance and preventive measures. It is important to note that our model reflects a moment in time, and does not show annual variation in tick distribution or how a future potential increase in temperatures may affect tick distribution and thus the potential for human exposure. Still, the study design is consistent between sites and repeatable, ensuring reliable future comparisons of tick distribution, and the produced maps allow for easy external validation. The resolution used to create our models may be too coarsely grained to catch small hotspots of tick presence/absence and the potential for human exposure. This is particularly evident for Denmark, which, throughout the country, has numerous small forest fragments smaller than 1 km^2^.

## Supplementary Data

797000 Supplement

## References

[1] Pfäffle M Littwin N Muders SV Petney TN The ecology of tick-borne diseases. Int J Parasitol. 2013;43(12-13):1059-77. 10.1016/j.ijpara.2013.06.009 23911308

[2] Jore S Vanwambeke SO Viljugrein H Isaksen K Kristoffersen AB Woldehiwet Z Climate and environmental change drives Ixodes ricinus geographical expansion at the northern range margin. Parasit Vectors. 2014;7(1):11. 10.1186/1756-3305-7-11 24401487 PMC3895670

[3] Skarphédinsson S Jensen PM Kristiansen K Survey of tickborne infections in Denmark. Emerg Infect Dis. 2005;11(7):1055-61. 10.3201/eid1107.041265 16022780 PMC3371797

[4] Jaenson TGT Jaenson DGE Eisen L Petersson E Lindgren E Changes in the geographical distribution and abundance of the tick Ixodes ricinus during the past 30 years in Sweden. Parasit Vectors. 2012;5(1):8. 10.1186/1756-3305-5-8 22233771 PMC3311093

[5] Jore S Viljugrein H Hofshagen M Brun-Hansen H Kristoffersen AB Nygård K Multi-source analysis reveals latitudinal and altitudinal shifts in range of Ixodes ricinus at its northern distribution limit. Parasit Vectors. 2011;4(1):84. 10.1186/1756-3305-4-84 21595949 PMC3123645

[6] Estrada-Peña A de la Fuente J The ecology of ticks and epidemiology of tick-borne viral diseases. Antiviral Res. 2014;108:104-28. 10.1016/j.antiviral.2014.05.016 24925264

[7] Medlock JM Hansford KM Bormane A Derdakova M Estrada-Peña A George J-C Driving forces for changes in geographical distribution of Ixodes ricinus ticks in Europe. Parasit Vectors. 2013;6(1):1-11. 10.1186/1756-3305-6-1 23281838 PMC3549795

[8] Andreassen A Jore S Cuber P Dudman S Tengs T Isaksen K Prevalence of tick borne encephalitis virus in tick nymphs in relation to climatic factors on the southern coast of Norway. Parasit Vectors. 2012;5(1):177. 10.1186/1756-3305-5-177 22913287 PMC3497858

[9] Lindgren E Gustafson R Tick-borne encephalitis in Sweden and climate change. Lancet. 2001;358(9275):16-8. 10.1016/S0140-6736(00)05250-8 11454371

[10] Public Health Agency of Sweden. TBE (Tick Borne Encephalitis) 2016. Solna: Folkhälsomyndigheten; 2017. Available from: https://www.folkhalsomyndigheten.se/folkhalsorapportering-statistik/statistikdatabaser-och-visualisering/sjukdomsstatistik/tick-borne-encephalitis-tbe/arsrapporter-och-kommentarer/2016/.

[11] Paulsen KM Pedersen BN Soleng A Okbaldet YB Pettersson JH-O Dudman SG Prevalence of tick-borne encephalitis virus in Ixodes ricinus ticks from three islands in north-western Norway. APMIS. 2015;123(9):759-64. 10.1111/apm.12412 26126504

[12] Soleng A Edgar KS Paulsen KM Pedersen BN Okbaldet YB Skjetne IEB Distribution of Ixodes ricinus ticks and prevalence of tick-borne encephalitis virus among questing ticks in the Arctic Circle region of northern Norway. Ticks Tick Borne Dis. 2018;9(1):97-103. 10.1016/j.ttbdis.2017.10.002 29030314

[13] Jaenson TGT Lindgren E The range of Ixodes ricinus and the risk of contracting Lyme borreliosis will increase northwards when the vegetation period becomes longer. Ticks Tick Borne Dis. 2011;2(1):44-9. 10.1016/j.ttbdis.2010.10.006 21771536

[14] Jaenson TGT Eisen L Comstedt P Mejlon HA Lindgren E Bergström S Risk indicators for the tick Ixodes ricinus and Borrelia burgdorferi sensu lato in Sweden. Med Vet Entomol. 2009;23(3):226-37. 10.1111/j.1365-2915.2009.00813.x 19712153

[15] Zeimes CB Olsson GE Hjertqvist M Vanwambeke SO Shaping zoonosis risk: landscape ecology vs. landscape attractiveness for people, the case of tick-borne encephalitis in Sweden. Parasit Vectors. 2014;7(1):370. 10.1186/1756-3305-7-370 25128197 PMC4143547

[16] European Centre for Disease Prevention and Control (ECDC). Country profile: Sweden. Tick-borne encephalitis (TBE). Stockholm: ECDC; 2012. Available from: https://ecdc.europa.eu/en/publications-data/country-profile-sweden-tick-borne-encephalitis-tbe

[17] Fomsgaard A Christiansen C Bødker R First identification of tick-borne encephalitis in Denmark outside of Bornholm, August 2009. Euro Surveill. 2009;14(36):1-2. 19758543

[18] Fomsgaard A Fertner ME Essbauer S Nielsen AY Frey S Lindblom P Tick-borne encephalitis virus, Zealand, Denmark, 2011. Emerg Infect Dis. 2013;19(7):1171-3. 10.3201/eid1907.130092 23764123 PMC3903456

[19] Randolph SE The shifting landscape of tick-borne zoonoses: tick-borne encephalitis and Lyme borreliosis in Europe. Philos Trans R Soc Lond B Biol Sci. 2001;356(1411):1045-56. 10.1098/rstb.2001.0893 11516382 PMC1088499

[20] Hvidsten D Stordal F Lager M Rognerud B Kristiansen B-E Matussek A Borrelia burgdorferi sensu lato-infected Ixodes ricinus collected from vegetation near the Arctic Circle. Ticks Tick Borne Dis. 2015;6(6):768-73. 10.1016/j.ttbdis.2015.07.002 26187417

[21] Lindström A Jaenson TGT Distribution of the common tick, Ixodes ricinus (Acari: Ixodidae), in different vegetation types in southern Sweden. J Med Entomol. 2003;40(4):375-8. 10.1603/0022-2585-40.4.375 14680099

[22] Landbo AS Flöng PT Borrelia burgdorferi infection in Ixodes ricinus from habitats in Denmark. Med Vet Entomol. 1992;6(2):165-7. 10.1111/j.1365-2915.1992.tb00596.x 1421487

[23] Jensen PM Hansen H Frandsen F Per Moestrup Jensen, Hanna Hansen Spatial risk assessment for Lyme borreliosis in Denmark. Scand J Infect Dis. 2000;32(5):545-50. 10.1080/003655400458857 11055662

[24] Mehl R The distribution and host relations of Norwegian ticks (Acari, Ixodides). Fauna Norv Ser B. 1983;31:46-58.

[25] Estrada-Peña A Farkas R Jaenson TGT Koenen F Madder M Pascucci I Association of environmental traits with the geographic ranges of ticks (Acari: Ixodidae) of medical and veterinary importance in the western Palearctic. A digital data set. Exp Appl Acarol. 2013;59(3):351-66. 10.1007/s10493-012-9600-7 22843316 PMC3557372

[26] Brownstein JS Holford TR Fish D A climate-based model predicts the spatial distribution of the Lyme disease vector Ixodes scapularis in the United States. Environ Health Perspect. 2003;111(9):1152-7. 10.1289/ehp.6052 12842766 PMC1241567

[27] Estrada-Peña A Geostatistics and remote sensing as predictive tools of tick distribution: a cokriging system to estimate Ixodes scapularis (Acari: Ixodidae) habitat suitability in the United States and Canada from advanced very high resolution radiometer satellite imagery. J Med Entomol. 1998;35(6):989-95. 10.1093/jmedent/35.6.989 9835691

[28] Sinka ME Rubio-Palis Y Manguin S Patil AP Temperley WH Gething PW The dominant Anopheles vectors of human malaria in the Americas: occurrence data, distribution maps and bionomic précis. Parasit Vectors. 2010;3(1):72. 10.1186/1756-3305-3-72 20712879 PMC2936890

[29] Elith J Leathwick JR Hastie T A working guide to boosted regression trees. J Anim Ecol. 2008;77(4):802-13. 10.1111/j.1365-2656.2008.01390.x 18397250

[30] Van Doninck J De Baets B Peters J Hendrickx G Ducheyne E Verhoest N Modelling the spatial distribution of Culicoides imicola: climatic versus remote sensing data. Remote Sens. 2014;6(7):6604-19. 10.3390/rs6076604

[31] Ducheyne E Miranda Chueca MA Lucientes J Calvete C Estrada R Boender GJ Abundance modelling of invasive and indigenous Culicoides species in Spain. Geospat Health. 2013;8(1):241-54. 10.4081/gh.2013.70 24258899

[32] Lühken R Gethmann JM Kranz P Steffenhagen P Staubach C Conraths FJ Comparison of single- and multi-scale models for the prediction of the Culicoides biting midge distribution in Germany. Geospat Health. 2016;11(2):405. 10.4081/gh.2016.405 27245797

[33] Peters J De Baets B Van Doninck J Calvete C Lucientes J De Clercq EM Absence reduction in entomological surveillance data to improve niche-based distribution models for Culicoides imicola. Prev Vet Med. 2011;100(1):15-28. 10.1016/j.prevetmed.2011.03.004 21496932

[34] Medley KA Niche shifts during the global invasion of the Asian tiger mosquito, Aedes albopictus Skuse (Culicidae), revealed by reciprocal distribution models. Glob Ecol Biogeogr. 2010;19(1):122-33. 10.1111/j.1466-8238.2009.00497.x

[35] Khatchikian C Sangermano F Kendell D Livdahl T Evaluation of species distribution model algorithms for fine-scale container-breeding mosquito risk prediction. Med Vet Entomol. 2011;25(3):268-75. 10.1111/j.1365-2915.2010.00935.x 21198711 PMC3135728

[36] Furlanello C, Neteler M, Merler S, Menegon S, Fontanari S, Donini A, et al. GIS and the random forest predictor: integration in R for tick-borne disease risk assessment. Proceedings of the 3rd International Workshop on Distributed Statistical Computing (DSC 2003).20-22 Mar 2003. Vienna, Austria. Available from: https://www.r-project.org/conferences/DSC-2003/Proceedings/FurlanelloEtAl.pdf

[37] Springer YP Jarnevich CS Barnett DT Monaghan AJ Eisen RJ Modeling the present and future geographic distribution of the lone star tick, Amblyomma americanum (Ixodida: Ixodidae), in the continental United States. Am J Trop Med Hyg. 2015;93(4):875-90. 10.4269/ajtmh.15-0330 26217042 PMC4596614

[38] Adalsteinsson SA, D’Amico V, Shriver WG, Brisson D, Buler JJ. Scale-dependent effects of nonnative plant invasion on host-seeking tick abundance. Peters DPC, editor. Ecosphere. 2016;7(3):e01317. 10.1002/ecs2.1317 PMC4827432 27088044

[39] Messina JP Pigott DM Golding N Duda KA Brownstein JS Weiss DJ The global distribution of Crimean-Congo hemorrhagic fever. Trans R Soc Trop Med Hyg. 2015;109(8):503-13. 10.1093/trstmh/trv050 26142451 PMC4501401

[40] Horobik V Keesing F Ostfeld RS Abundance and Borrelia burgdorferi-infection prevalence of nymphal Ixodes scapularis ticks along forest–field edges. EcoHealth. 2006;3(4):262-8. 10.1007/s10393-006-0065-1

[41] Jackson LE Hilborn ED Thomas JC Towards landscape design guidelines for reducing Lyme disease risk. Int J Epidemiol. 2006;35(2):315-22. 10.1093/ije/dyi284 16394113

[42] Glass GE Schwartz BS Morgan JM 3rd Johnson DT Noy PM Israel E Environmental risk factors for Lyme disease identified with geographic information systems. Am J Public Health. 1995;85(7):944-8. 10.2105/AJPH.85.7.944 7604918 PMC1615529

[43] Eisen RJ Lane RS Fritz CL Eisen L Spatial patterns of Lyme disease risk in California based on disease incidence data and modeling of vector-tick exposure. Am J Trop Med Hyg. 2006;75(4):669-76. 10.4269/ajtmh.2006.75.669 17038692

[44] MODIS v5: Temporal Fourier Analysis (TFA). Imagery update 2001-12. PALE-Blu Data Portal; 2014. Available from: https://www.edenextdata.com/?q=content/modis-v5-temporal-fourier-analysis-tfa-imagery-update-2001-12

[45] Corine land cover 2006 raster data. Copernicus programme; 2010. Available from: https://www.eea.europa.eu/data-and-maps/data/clc-2006-raster

[46] R Development Core Team. R: A Language and Environment for Statistical Computing. Vienna: R Foundation for Statistical Computing; 2017. Available from: http://www.R-project.org/

[47] Socioeconomic Data and Applications Center. Gridded Population of the World (GPW), v4. New York: Columbia University. [Accessed: 12 Aug 2017. Available from: http://sedac.ciesin.columbia.edu/data/collection/gpw-v4

[48] Bernes C. Biologisk mångfald i Sverige. [Biological diversity in Sweden]. Stockholm: Naturvårdsverket; 2011. Swedish.

[49] Hijmans RJ Cameron SE Parra JL Jones PG Jarvis A Very high resolution interpolated climate surfaces for global land areas. Int J Climatol. 2005;25(15):1965-78. 10.1002/joc.1276

[50] Food and Agriculture Organization (FAO), International Institute for Applied Systems Analysis (IIASA), ISRIC-World Soil Information, Institute of Soil Science, Chinese Academy of Sciences, Joint Research Centre of the European Commission. Harmonized world soil database v 1.2. Rome: FAO and Laxenburg: IIASA; 2009.

