# Supplementary material for: Predicting and mapping human risk of exposure to Ixodes ricinus nymphs using climatic and environmental data, Denmark, Norway and Sweden, 2016
Source: Euro Surveill. 2019 Feb 28;24(9):1800101. doi: 10.2807/1560-7917.ES.2019.24.9.1800101 (PMC6402176; doi:10.2807/1560-7917.ES.2019.24.9.1800101)

This supplementary material is hosted by *Eurosurveillance* as supporting information alongside the article "Predicting and mapping human risk of exposure to *Ixodes ricinus* nymphs using climatic and environmental data, Denmark, Norway and Sweden, 2016" on behalf of the authors who remain responsible for the accuracy and appropriateness of the content. The same standards for ethics, copyright, attributions and permissions as for the article apply. *Eurosurveillance* is not responsible for the maintenance of any links or email addresses provided therein.

# Predicting and mapping human risk of exposure to *Ixodes ricinus* nymphs using climatic and environmental data, Denmark, Norway and Sweden, 2016 - Supplementary material

## Environmental variables

We used environmental data derived from images from a MODIS satellite v5 time series for 2001-2012. The images were processed applying a Temporal Fourier Analysis (TFA) [1], where environmental cycles (temperature, vegetation phenology etc.) are described as the sum of a series of sine curves with different amplitudes and phases. The MODIS data were composited over 8- or 16-day time intervals, and the TFA applied used a spline-based algorithm developed by Scharlemann et al. [1]. We used the TFA processed raster images for the middle infra-red index (channel 03), daytime land surface temperature (channel 07), nighttime land surface temperature (channel 08), the normalized difference vegetation index (NDVI, channel 14), and the enhanced vegetation index (EVI, channel 15). We used the following Fourier processed outputs for each of the variables: mean, minimum, maximum, variance in raw data, combined variance in annual, bi-annual, and tri-annual cycles as well as amplitude, phase and variance of annual bi-annual and tri-annual cycle [2]. For MODIS code of the Fourier outputs, see Supplementary Table S1. This dataset was created and processed by the TALA research group of Oxford University and obtained through the EDENext project [2].

We also used average monthly climate data from Worldclim and Bioclim (1960 to 1990). These datasets provided us with global raster files of altitude and of temperature and precipitation. The Bioclim images further gave us information on annual trends such as mean annual temperature and annual precipitation as well information on seasonality in the form of annual ranges and quarterly temperatures and precipitation (for example temperature of the coldest and warmest month, and precipitation of the wettest and driest quarters) [3].

The Corine land cover (CLC) is a raster image of 44 land cover classes created for 12 European countries, and comes in resolutions from 100 $m^2$ to 1$km^2$. We obtained this data from the European Environment Agency website [4].

Lastly, we used data on soil types obtained from the Harmonized World Soil Database v1.2 from 2009 [5].

## Stratification of study region

We divided each of the three countries into a north and a south region (with equal areas in the north and south), and used the Corine land cover data [4] to divide the resulting six regions into forest, meadow and others. We defined forest as the Corine land cover types: Broad-leaved forest, Coniferous forest and Mixed forest (135,996 $km^2$) and defined meadow as the Corine land cover types: Land principally occupied by agriculture with significant areas of natural vegetation, Natural grasslands, Moors and heathland, and Transitional woodland-shrub (21,336 $km^2$). All other land cover classes e.g. urban and agricultural areas were classified as 'others' and not sampled for ticks or predicted by the resulting model. We used the Fourier transformed satellite data for maximum NDVI (maximum values for the time series) [2]. We used maximum NDVI values for each $km^2$ in the region and calculated the median value for each of the six regions. We then

defined low maximum NDVI as all values below or equal to the median and everything above as high maximum NDVI, resulting in 8 strata for each country (Supplementary Table S2 and S3 and Figure 1 in manuscript). We used the NDVI index as it is based on the relationship between red light and near infrared light (NIR). Reflected red energy decreases with plant development due to the chlorophyll absorption within actively photosynthetic leaves. Reflected NIR energy, on the other hand, will increase with plant development through scattering processes in healthy leaves, thus the NDVI index gives us information on the amount of vegetation at a given site.

## Boosted Regression tree modelling

We used packages caret [6] and gbm (generalized boosted regression models) [7] in R 3.4.2. to create a boosted regression tree model (BRT). The boosting method used in the gbm package follows Friedman's Gradient Boosting Machine [8,9], and iteratively adds basis functions (i.e. small trees) in a greedy fashion to reduce the selected loss function using steepest descent. The chosen loss function was deviance, assuming our data to be Bernoulli distributed (logistic regression for 0-1 outcome). Within each tree, the split criterion was determined using Friedman's mean square error (MSE)[8,10].

Performance of a BRT model may be heavily influenced by class imbalance [11]. As our data consisted of 79% presences and 21% absences, we investigated different balancing methods (no balancing, down-scaling, up-scaling, rose (package rose in R), smote [11] and tomek [11]). The CV scheme was carried out for each balancing method, and was run 10 times with different random seeds to choose the best-performing balancing method according to highest score for the area under the curve (AUC) for the receiver operating characteristic (ROC) [12]. With 125 presence and 34 absence points (Table 2 in Manuscript), tomek-balancing, where the majority class was removed, consistently gave a higher AUC for the 10 different seeds (1-way ANOVA: $F_{6,63} = 36.07$, $p < 0.001$, Supplementary Figure S1), and this balancing method was therefore chosen for the final model. We performed stratified fivefold cross-validation with 10 repetitions to validate our models and to estimate the prediction error. A tuning grid was used to optimise model parameters, interaction depth, number of trees, learning rate and minimum observations per node [12]. Using the tuning grid, the final model had the following parameters: 1,500 trees, an interaction depth of 1, a learning rate of 0.01 and a minimum number of observations of 3 per node. When evaluating the model over the different folds and repeats, the accuracy was 0.85 with a sensitivity of 91%, a specificity of 60% (given a fixed cut-off of 50% PP) and an AUC-score of 0.86 (Supplementary Figure S2).

## Population density maps

Using the Gridded Population of the World dataset [13], we first created nine new rasters from the final prediction map, that only included forest and meadow pixels where the probability of presence (PP) was higher or equal to 10%, 20%, 30%, 40%, 50%, 60%, 70%, 80% and 90% respectively. For each of the nine rasters, we calculated the Euclidian Distance with Spatial Analyst Tools in ArcMap [14] to create five additional rasters, depicting pixels within a distance of 1, 2, 3, 4, and 5 km respectively to forest/meadow pixels (5 rasters for each PP cut-off). We finally cropped the human population density raster, to only encompass raster pixels within those distances of 1-5 km from forest and meadow (5x9 rasters, these could include people living above 450 meters of altitude, if they lived within 5 km of forest/meadows at altitudes below 450 m). For each of the 45 rasters we calculated the percentage of people living within the pixels out of the total number of people in the modelled region.

## References


1. Scharlemann JPW, Benz D, Hay SI, Purse B V., Tatem AJ, Wint GRW, et al. Global Data for Ecology and Epidemiology: A Novel Algorithm for Temporal Fourier Processing MODIS DataPLoS One. 2008;3(1): e1408. http://dx.plos.org/10.1371/journal.pone.0001408

2. MODIS v5: Temporal Fourier Analysis (TFA). Imagery update 2001-12. PALE-Blu Data Portal; 2014. Available from: https://www.edenextdata.com/?q=content/modis-v5-temporal-fourier-analysis-tfa-imagery-update-2001-12

3. Hijmans RJ, Cameron SE, Parra JL, Jones PG, Jarvis A. Very high resolution interpolated climate surfaces for global land areas. Int J Climatol. 2005;25(15):1965-1978. doi:10.1002/joc.12764.

4. Corine land cover 2006 raster data. Copernicus programme. 2010. Available from: https://www.eea.europa.eu/data-and-maps/data/clc-2006-raster

5. Harmonized world soil database v1.2 2009. Available from: http://www.fao.org/soils-portal/soil-survey/soil-maps-and-databases/harmonized-world-soil-database-v12/en/.

6. Kuhn M. Contributions from Jed Wing and Steve Weston and Andre Williams and Chris Keefer and Allan Engelhardt and Tony Cooper and Zachary Mayer and Brenton Kenkel and the R Core Team and Michael Benesty and Reynald Lescarbeau and Andrew Ziem and Luca Sc. caret: Classification and Regression Training. R package version 6.0-78. 2017.

7. Ridgeway G. with contributions from others. gbm: Generalized Boosted Regression Models. R package version 2.1.3. 2017. Available from: https://cran.r-project.org/package=gbm

8. Friedman JH. Greedy function approximation: A gradient boosting machine. Ann Stat. 2001;29:1189–232.

9. Ridgeway G. Generalized Boosted Models: A guide to the gbm package. 2007. Available from: http://www.saedsayad.com/docs/gbm2.pdf

10. Friedman JH. Stochastic gradient boosting. Comput Stat Data Anal. 2002;38(4):367-378. doi:10.1016/S0167-9473(01)00065-2

11. Batista GEAPA, Prati RC, Monard MC. A Study of the Behavior of Several Methods for Balancing Machine Learning Training Data. ACM SIGKDD Explor Newsl. 2004;6(1):20–29. doi:10.1145/1007730.1007735

12. Elith J, Leathwick JR, Hastie T. A working guide to boosted regression trees. J Anim Ecol. 2008;77(4):802-813. doi:10.1111/j.1365-2656.2008.01390.x

13. Gridded Population of the World, v4. Socioeconomic Data and Applications Center (SEDAC). 2015. Available from: http://sedac.ciesin.columbia.edu/data/collection/gpw-v4

14. ESRI 2011. ArcGIS Desktop: Release 10.1 Redlands, CA: Environmental Systems Research Institute.

## Tables

**Supplementary Table S1. MODIS codes for the outputs, resulting from Fourier processing of different environmental variables derived from MODIS satellite images.**

| Fourier output | Modis code for Fourier output |
|---|---|
| mean | a0 |
| minimum | mn |
| maximum | mx |
| amplitude of annual cycle | a1 |
| amplitude of bi-annual cycle | a2 |
| amplitude of tri-annual cycle | a3 |
| phase of annual cycle | p1 |
| phase of bi-annual cycle | p2 |
| phase of tri-annual cycle | p3 |
| variance in annual cycle | d1 |
| variance in bi-annual cycle | d2 |
| variance in tri-annual cycle | d3 |
| combined variance in annual, bi-annual, and tri-annual cycles | da |
| variance in raw data | vr |

**Supplementary Table S2. All first priority sample sites in each of Denmark, Norway and Sweden, 2016**

| Region | Strata | No. of 1st Priority sample sites in each country |
|---|---|---|
| North | Forest, low NDVI | 6 |
| | Forest, high NDVI | 6 |
| | Non-forest, low NDVI | 2 |
| | Non-forest, high NDVI | 1 |
| South | Forest, low NDVI | 6 |
| | Forest, high NDVI | 6 |
| | Non-forest, low NDVI | 1 |
| | Non-forest, high NDVI | 2 |

**Supplementary Table S3. All first priority sample sites around the Oslo Fjord in Norway, 2016**

| Region | Strata | No. of 1st Priority sample sites |
|---|---|---|
| North | Forest, low NDVI | 4 |
| | Forest, high NDVI | 4 |
| | Non-forest, low NDVI | 1 |
| | Non-forest, high NDVI | 1 |
| South | Forest, low NDVI | 4 |
| | Forest, high NDVI | 4 |
| | Non-forest, low NDVI | 1 |
| | Non-forest, high NDVI | 1 |

## Figures

**Supplementary Figure S1. Area Under the Curve (AUC) of the original data and the different balancing methods down, up, rose, smote and tomek with majority class removed (tomek_maj), and tomek with both classes removed (tomek_both). The AUC-scores were obtained by re-running the cross-validation for each balancing methods with 10 different random seeds (chosen seeds were similar for all balancing methods).**

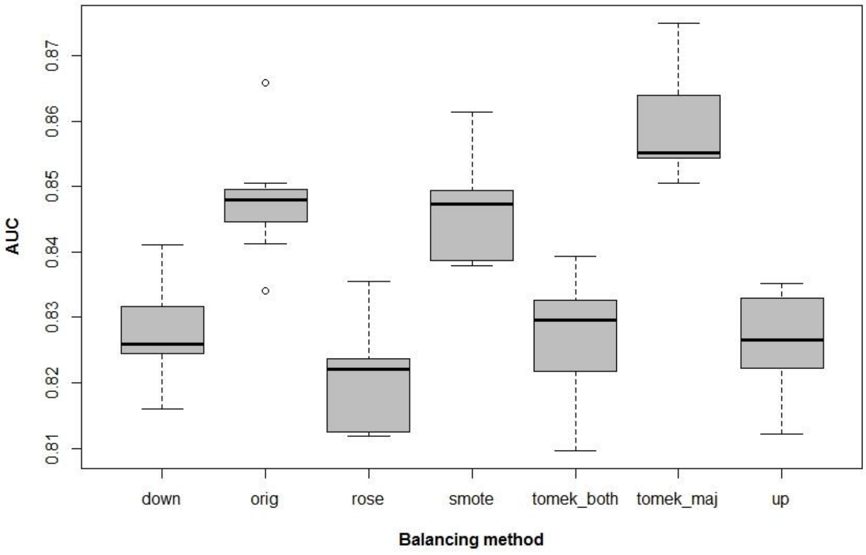


**Supplementary Figure S2. Receiver Operating Curve curve and Area Under the Curve for the final boosted regression tree model (using the balancing method tomek with the majority class removed), used to create prediction maps of nymphal *I. ricinus* distribution in southern Scandinavia.**

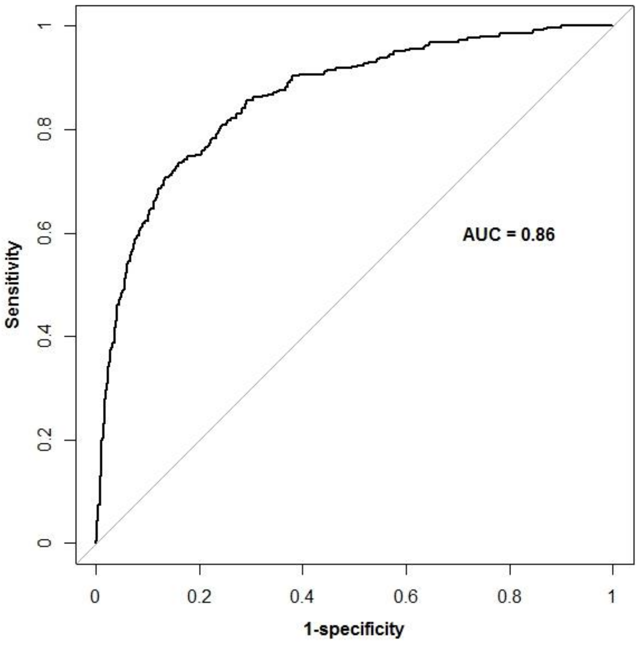

**Supplementary Figure S3. Prediction errors for the predicted values (probability of presence, PP) – observed values (0 or 1 for absence/presence), based on the final boosted regression tree model to predict nymphal *I. ricinus* distribution in southern Scandinavia. High negative values show sites with high PP, but with measured absence of tick nymphs, whereas high positive values indicate sites with low PP, but with measured presence of tick nymphs. White areas within Denmark, Norway and Sweden are altitudes above 450 m or lakes, rivers and streams.**

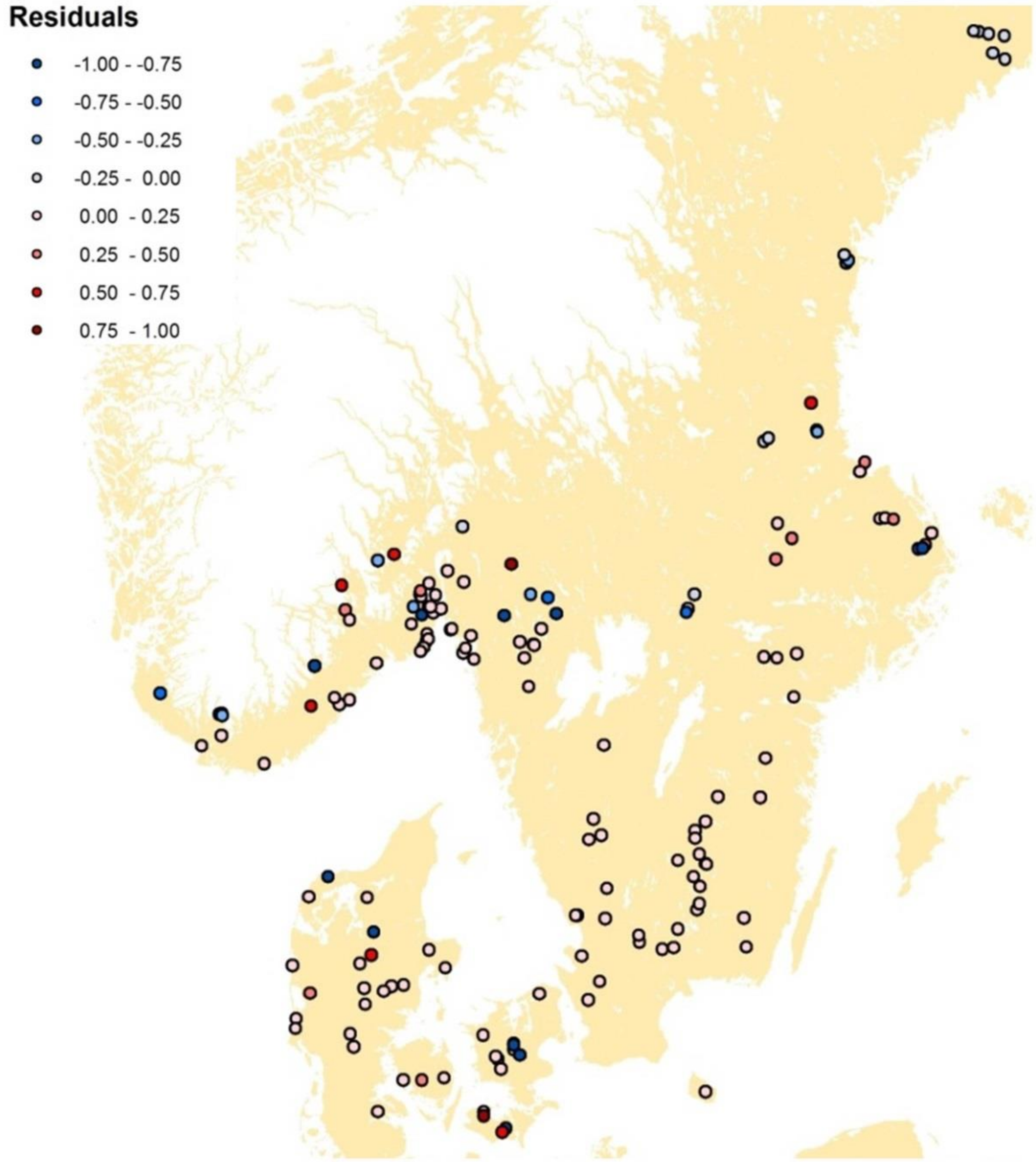

**Supplementary Figure S4. Plots of the 5 most important predictors in the final boosted regression tree model predicting nymphal *I. ricinus* distribution in southern Scandinavia. All are partial dependence plots, except the land cover plot, and illustrates the marginal effect of the selected variables on the response after integrating out the other variables. As the boosted regression tree model produces dummy variables for factorial predictors, we cannot show partial dependence plots for Corine land cover (in our model, only land cover type 29, "Transitional woodland-shrub", was one of the top 5 predictors). Instead, the plot shows probability of presence plotted against the different land cover types in our model.**

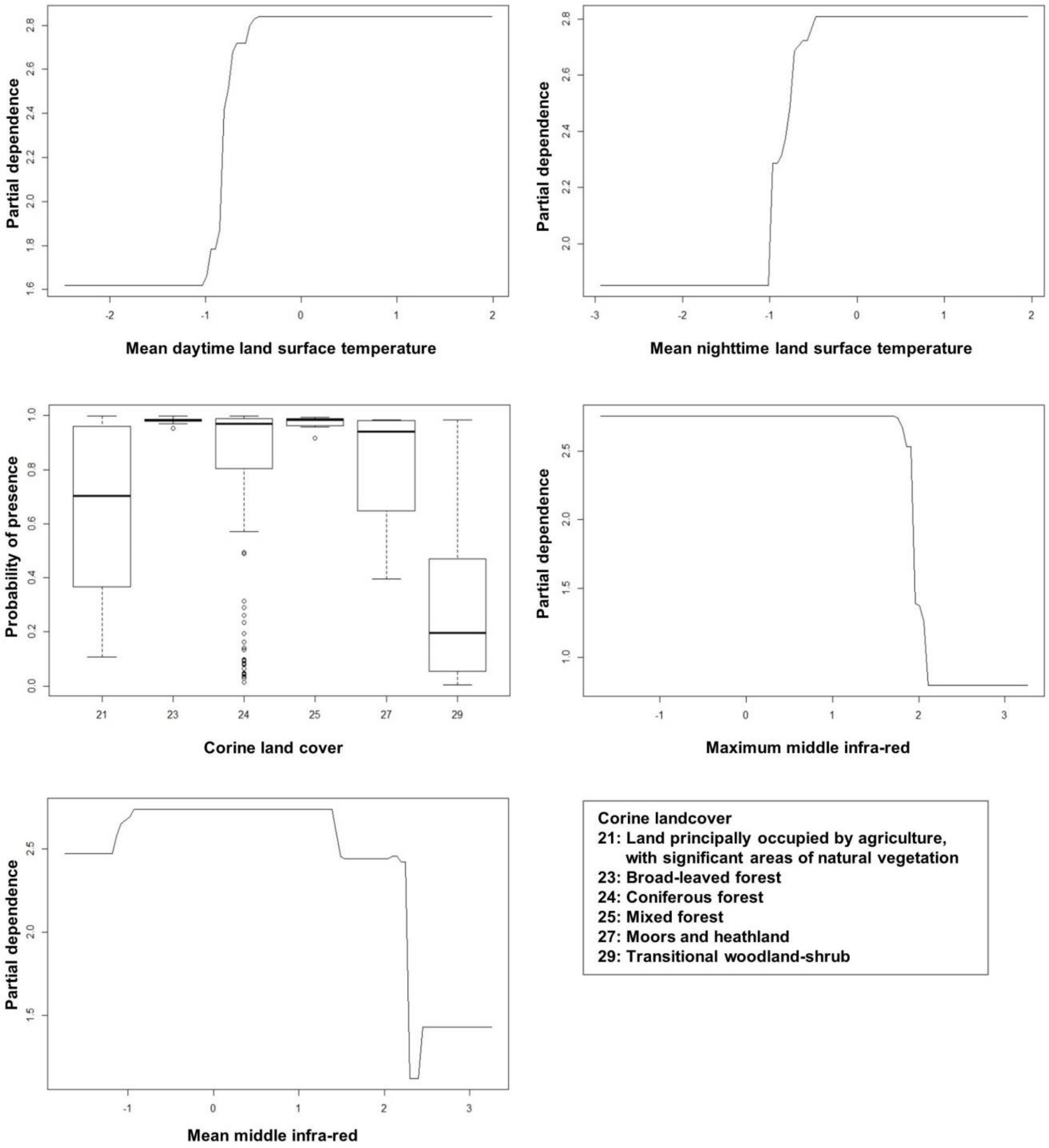

**Supplementary Figure S5. Map of mean annual temperature in Scandinavia (BIO1 from BioClim, see Table 1 in the manuscript). Temperature is in the format °Cx10.**

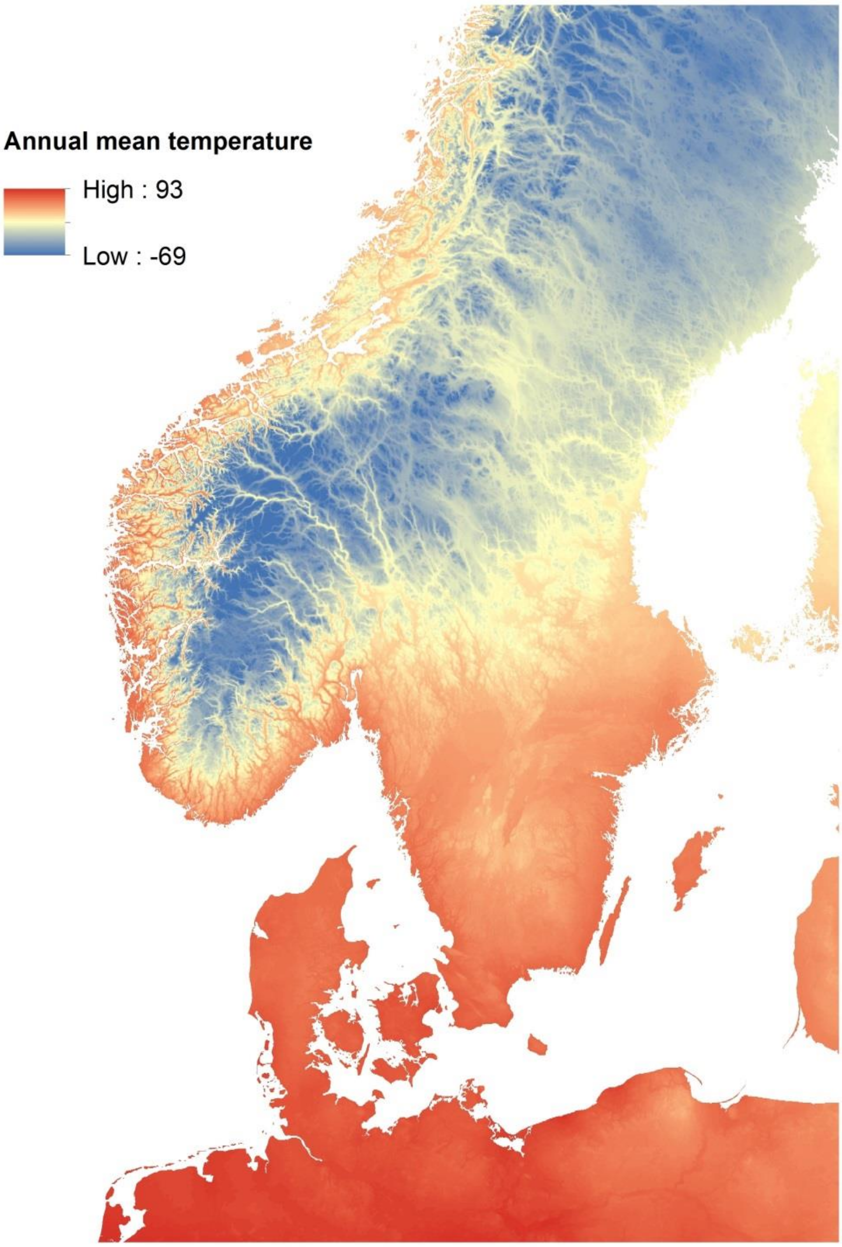

Supplement: Supplement [file 1800101_KJAER_Supplement.pdf]
